# One-Step Mask Etching Strategy Toward Ordered Ferroelectric Pb(Zr_0.52_Ti_0.48_)O_3_ Nanodot Arrays

**DOI:** 10.1186/s11671-015-1028-7

**Published:** 2015-08-07

**Authors:** Xiaoyan Zhang, Mengyang Kang, Kangrong Huang, Fengyuan Zhang, Sixian Lin, Xingsen Gao, Xubing Lu, Zhang Zhang, Junming Liu

**Affiliations:** Institute for Advanced Materials and Guangdong Provincial Key Laboratory of Quantum Engineering and Quantum Materials, South China Academy of Advanced Optoelectronics, South China Normal University, Guangzhou, 510006 China; Laboratory of Solid State Microstructures and Innovation Center of Advanced Microstructures, Nanjing University, Nanjing, 210093 China

**Keywords:** AAO, PZT, Barrier layer, Mask etching, Nanodot arrays

## Abstract

In this report, ordered lead zirconate titanate Pb(Zr_0.52_Ti_0.48_)O_3_ (PZT) nanodot arrays were fabricated by an original one-step mask etching route. The one-step mask etching strategy is based on the patterned nanostructure of barrier layer (BL) at the bottom of anodic aluminum oxide (AAO), by a direct transfer of the nanopattern from BL to the pre-deposited PZT film, without introduction of any sacrifice layer and lithography. Therefore, the presented strategy is relatively simple and economical. X-ray diffraction and Raman analysis revealed that the as-prepared PZT was in a perovskite phase. Atomic and piezoresponse force microscopy indicated that the PZT nanodot arrays were with both good ordering and well-defined ferroelectric properties. Considering its universality on diverse substrates, the present method is a general approach to the high-quality ordered ferroelectric nanodot arrays, which is promising for applications in ultra-high density nonvolatile ferroelectric random access memories (NV-FRAM).

## Background

Ordered nanodot arrays have attracted great research attention in the last decade because of their applications in the functional nanodevices, such as high-density information storage media [1, 2] and highly sensitive sensors [3, 4]. For the metallic and semiconductor nanomaterials, the most used manufacture technique was lithography [5, 6]. Nevertheless, for complex materials such as ternary/quaternary perovskite oxides, it has been proven to be an inappropriate route by lithography as the etching process can render a damaged region at the rim of the patterned nanostructure [7, 8]. Molecular beam epitaxy (MBE) [9-11] and self-organized growth techniques [12, 13] were more appropriate than the complicated lithography. However, these techniques are limited by the long assembly time, more dislocation defects, destructive crystal structure, long-range ordering, and short-range disordering [8, 14]. Therefore, it is necessary to exploit fabrication of high-quality ordered nanodot arrays over large areas with more universal and practical approaches.

Nanopatterning based on multi-step mask etching process offers plenty of advantages to produce ordered arrays of nanostructures as compared to the typical lithographic methods, such as the ability to pattern over large areas simultaneously and relatively low costs [15]. Since Masuda et al*.* reported the two-step anodization method for self-organized nonporous anodic aluminum oxide (AAO) [16, 17], the low-dimensional nanostructures using AAO templates have drawn increasing interests. In particular, AAO templates are with advantages of large-area fabrication, paralleled pore arrangement, tunable pore diameter, pore length, interpore distance, and good pore ordering [18]. Pore-through thin AAO membrane can be also used as a nanopatterning mask, combined with many other techniques, to synthesize functional nanodot arrays. These techniques include chemical vapor deposition (CVD) [19], physical vapor deposition (PVD) [20, 21], and dry etching [22–29]. The conventional dry etching techniques mainly include plasma etching (PE), reactive ion etching (RIE), and ion beam etching (IBE). PE and RIE need different kinds of excitation conditions and dangerous reactive gases, such as the fluoride and chloride [22–24]. IBE is a physical etching process using Ar gas as the ion source, and it has the advantages of human safety, anisotropic etching, and less line width loss when the feature size is less than 1 μm [24–29]. Typically, the use of AAO mask required the removal of the barrier layer (BL), in order to realize a pore-through structure by selective chemical etching or IBE [29, 30]. BL was a thin textured aluminum oxide (Al_2_O_3_) film at the interface between AAO and Al. In the BL, the hemispherical cavities at each pore bottom of AAO are also arranged in a hexagonal close-packed geometry. And the hemispherical cavity arrays are even with a better uniformity and ordering than the pores of AAO [18]. Obviously, the established uses of AAO mask have disadvantages in the practical applications, such as the detriment to the pore uniformity and the inevitable pore enlargement effect [31]. In the AAO template-assisted growth, after getting rid of the template to leave the as-grown nanostructures, the stress release always resulted in the degradation of the device performance [20, 21]. Otherwise, by the multi-step mask etching, in order to obtain the ordered nanodot arrays on the functional film, a sacrificial layer is prerequisite in the middle of the AAO and the film. Complicated lithographs are still involved to first transfer the nanopattern from the AAO to the sacrifice layer [6, 12].

Obviously, the multi-step mask etching process with pore-through AAO is still complicated and time-consuming. However, to our best knowledge, the use of AAO with BL as mask to directly transfer the nanopattern to the functional film has not been reported. In this work, an original one-step mask etching strategy to fabricate ordered ferroelectric nanodot arrays is present, using the BL-connected AAO as mask. The fabricated lead zirconate titanate Pb(Zr_0.52_Ti_0.48_)O_3_ (PZT) nanodot arrays showed controllable size, good ordering, high-quality crystallinity, and well-defined ferroelectric properties. Considering the universality of the IBE on diverse substrates, the present method is a general approach to the high-quality ordered ferroelectric nanodot arrays, which is promising for applications in ultra-high density nonvolatile ferroelectric random access memories (NV-FRAM).

## Methods

### AAO Fabrication

In order to obtain AAO with a good ordering, first, high-purity aluminum foils (99.999 %, Goodfellow Cambridge Limited) were annealed at 450 °C for 3 h in Ar atmosphere, before electropolishing in a mixture of HClO_4_ and C_2_H_5_OH (1:3 by volume) with a constant voltage of 20 V for 5 min. Then, a standard two-step anodization method was used, by using phosphoric acid as electrolyte with a corresponding constant anodization voltage of 195 V. The first anodization lasted for at least 24 h, and then the oxide layer was completely removed by a wet chemical etching (a mixture of 1.8 wt.% chromic acid and 6 wt.% phosphoric acid) at 50 °C to obtain a textured surface on Al. The second anodization was conducted with the same electrochemical parameters as the first, with a 300-s oxidation. In order to obtain AAO membrane bonded on the Pt/Si substrate, firstly, a thin layer of polystyrene (PS) (1 wt.% PS/CHCl_3_ solution) was spin-coated onto the top of AAO film, followed by a 90 °C solidification heating. After that, a CuCl_2_ solution (6.8 g CuCl_2_ + 100 ml 37 % HCl + 200 ml distilled water) was used to remove the Al foil on the back side of the AAO. Then, the AAO film was directly transferred to the substrate without extra pore-widening and pore-through processes.

### Sol-Gel Synthesis of PZT Film

The nanocomposite PZT thin film was prepared by a sol-gel process and spin coating technique. A 0.4 M PZT sol-gel precursor with the molar ratio of 1:0.52:0.48 was prepared by dissolving zirconium propoxide (Zr(CH_2_CH_2_CH_3_O)_4_) and isopropyl titanate (Ti(C_4_H_9_O)_4_) into 2-methoxyethanol (C_3_H_8_O_2_), with acetic acid and propanol added as the solvents, and stirring until completely dissolved. Then, 10 % excess lead acetate trihydrate (Pb(CH_3_CO_2_)_2_·3H_2_O) was added with the purpose of compensating the lead loss and preventing forming the second phase of pyrochlore-type in the annealing process [32]. The precursor solution was stirred for 24 h at room temperature and then aged for 1 week. Before the spin coating, the prepared solution was diluted to 0.1 M. The diluted solution was spin-coated onto Pt/Si substrate with a thickness of 0.5 mm at 4000 rpm for 90 s and subsequently baked at 120 and 350 °C for 5 min each. This spin coating and baking procedure was repeated twice on a hot plate to evaporate the solvents. After being calcined at 700 °C for 20 min in air in rapid thermal process (RTP), a nanocomposite PZT film with a thickness of 100 nm was obtained.

### IBE Process

The specimen was etched by Ar ion beam etching (MIBE-150C) in an ambient pressure of 5.6 × 10^−4^ mbar at room temperature. The total etching time was about 30 min to form the optimum PZT nanodot arrays. The whole etching process was with a vertical incident ion beam to the substrate. The etching energy was set to a cathode current of 11.5 A, anode voltage of 55 V, plate voltage of 300 V, ion accelerating voltage of 250 V, neutralization current of 13 A, and bias current of 1.2 A. After the IBE process, the remaining AAO mask was completely removed off by dipping the specimen into phosphoric acid.

### Characterizations

The nanostructure characterization was carried out ex situ with a field-emission scanning electron microscope (FE-SEM; Zeiss Ultra 55). Atomic/piezoresponse force microscopy (AFM/PFM; Cypher™, Asylum Research) was used to characterize the surface morphologies and the ferroelectric properties of PZT nanodot arrays. The crystallization properties of the PZT nanodots were investigated using X-ray diffraction (XRD; Philips X’Pert Pro) with an X-ray wavelength of 0.15406 nm. The Raman spectrums were measured at room temperature by the inVia Reflex Raman microscope (Renishaw Corp.) with an excitation wavelength of 633 nm, and the laser power was set to 10 % of its maximum.

## Results and Discussion

### One-Step Mask Etching

**Fig. 1:**
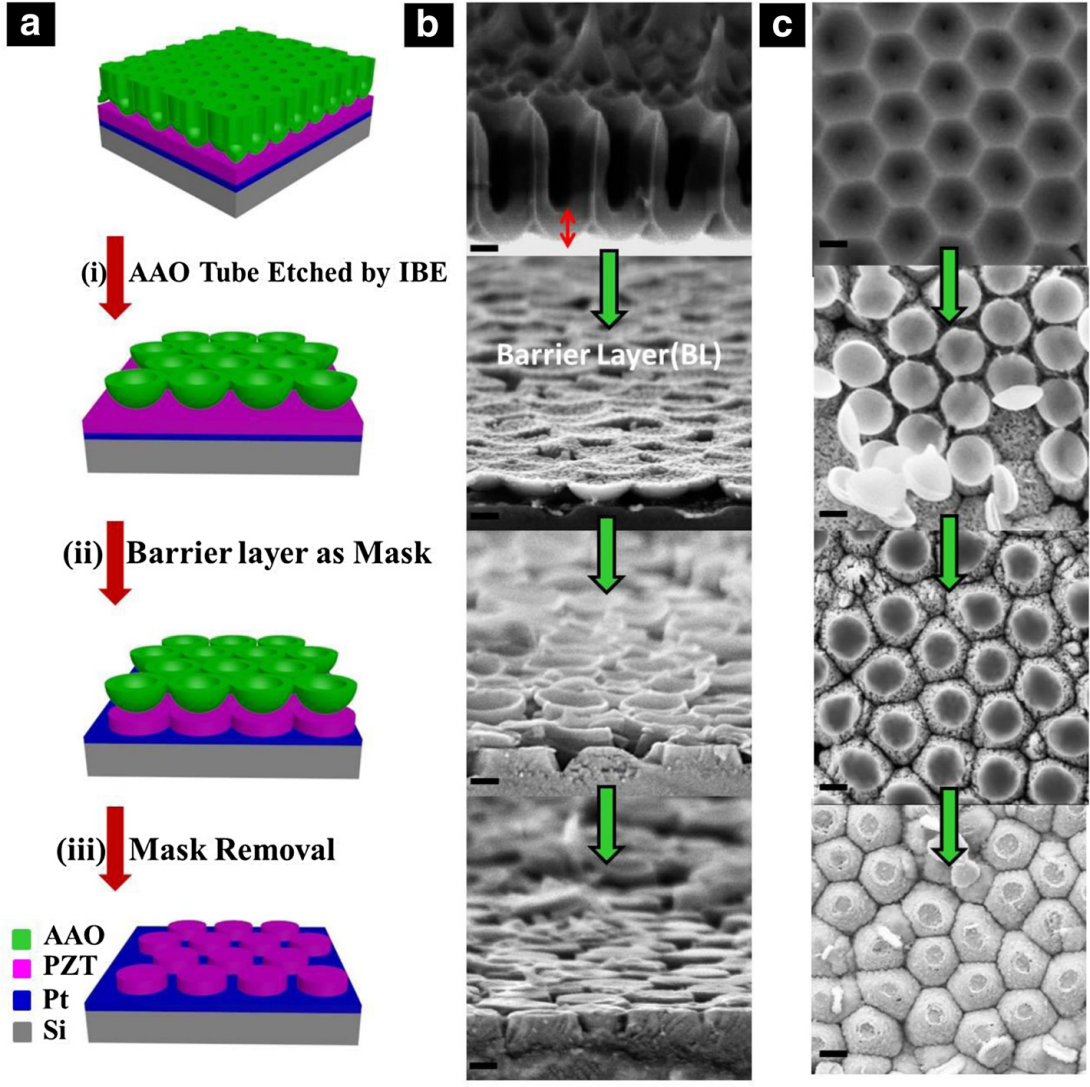
Fabrication details for the PZT nanodot arrays. **a** Schematic illustration of the fabrication procedures of ordered PZT nanodot arrays by one-step mask etching method, utilizing the barrier layer of AAO as mask: (*i*) the first long-time etching by the Ar IBE to remove the above porous structure of AAO, (*ii*) the second etching of short time with the barrier layer as mask, and (*iii*) removal of the remaining barrier layer. Corresponding tilted side-view and top-view SEM images of each step are shown in **b** and **c**, respectively. *Scale bars* are 200 nm

The one-step mask etching process was schematically illustrated in Fig. 1a. First, without the pore-widening and pore-through procedures, the AAO membrane with BL was directly transferred onto the PZT thin film, which was synthesized on the Pt/Si substrate by a sol-gel method. Subsequently, the AAO tubular nanostructure was gradually etched off by IBE from top to bottom in about 25 min, resulting in the disconnected hemispherical bowl-like BL nanostructures remained on the PZT film (Fig. 1a(i)). Then, the IBE was proceeded for an extra 5 min to transfer the nanopattern of BL mask to the PZT film (Fig. 1a(ii)). Finally, after the complete removal of the remaining BL on the PZT, the ordered PZT nanodot arrays sitting on a conductive Pt/Si substrate were obtained (Fig. 1a(iii)). During the IBE process, the neutral-charged Ar ion beam transmitted its kinetic energy to the atoms of the specimen to sputter out. The high-powered Ar ion beam can also play the role of dustman to clean the sputtering redeposition on surfaces to speed up the etching process.

The tilted side-view and top-view SEM images were shown in Fig. 1b, c, respectively, corresponding to each etching step from top to bottom (illustrated in Fig. 1a). Due to the bombarding effect of Ar ion beam, the tubular nanostructure of AAO was first etched away, leaving the disconnected hemispherical bowl-like BL nanostructures on the surface of the PZT film. Actually, the ordered nanostructures of BL played the role of etching mask for the nanopatterning on the PZT film. Since the round edges of the hemispherical BL were preferentially etched, the PZT film was patterned according to the interspaces between the mask with wedge-shaped grooves. With its diameter decreasing, the BL was also etched to be thinner, being transformed from the initial hemispherical bowl-like nanostructure to the flat nanoplate (shown in the middle two of Fig. 1b, c). After removal of the BL, the trapezoid cylinder-shaped PZT nanodot arrays were formed on the Pt/Si substrate. The diameter, inter-dot distance, and density of the PZT nanodot was about 400 nm, 500 nm, and 10^9^ cm^−2^, respectively, with the exactly the same ordering as the AAO. The hexagonal bottom edges of PZT nanodots shown in the top-view morphology in Fig. 1c (bottom one) was mainly according to the boundary void shape of hexagonally close-packed AAO tubes [18]. The slight deformation of the hexagonal edges was probably caused by the different etching rates corresponding to the deviation of incident beam angles, on account of the cascade collision model of the sputtered atoms [26–28].

Obviously, the whole etching process is without introduction of any sacrifice layer and lithography process, which is a typical one-step etching process. Its capability of nanopatterning ordered nanodot arrays on the PZT film is quite different from the conventional one-step mask etching process that produced nanohole arrays by the RIE or PE [12, 34].

### Phase Analysis

**Fig. 2:**
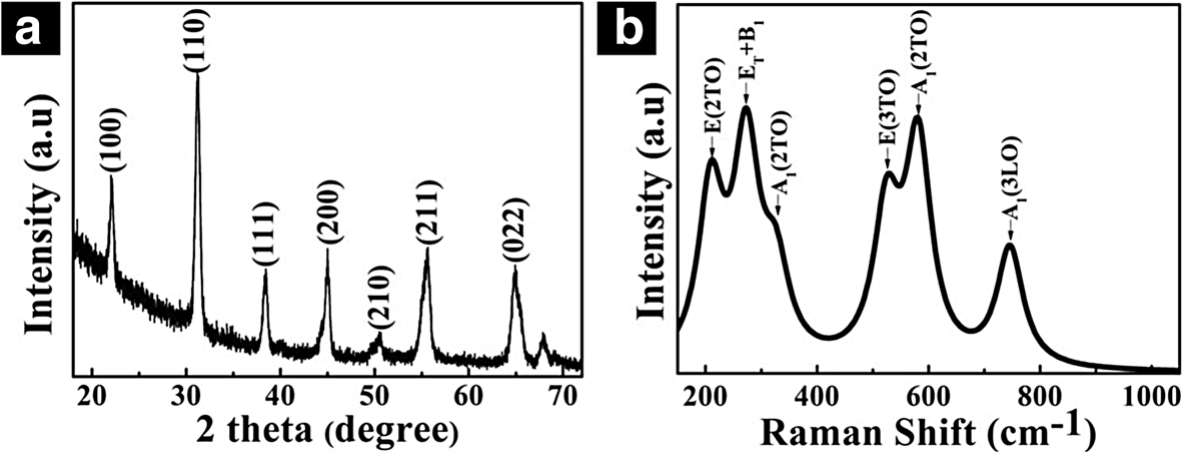
X-ray diffraction pattern and Raman spectrum of the ordered PZT nanodot arrays measured at room temperature. **a** X-ray diffraction pattern and **b** Raman spectrum

Figure 2a, b illustrated the X-ray diffraction pattern (XRD) and Raman scattering spectrum of PZT nanodot arrays, respectively, measured at room temperature. Generally, the as-deposited PZT film was amorphous, and a post-deposition annealing was needed to transform the film from the amorphous to the desirable ferroelectric perovskite phase. The amorphous structure will first transform into an intermediate pyrochlore phase which was not expected in the final phase, and then the pyrochlore phase will transform into the perovskite phase higher than 650 °C. Actually, the perovskite phase grew from the surface of the pyrochlore film [32]. Figure 2a shows the XRD pattern of the PZT nanodot arrays fabricated by the one-step mask etching strategy; to avoid the pyrochlore phase, the annealing temperature was set to 700 °C and 10 % excess lead acetate trihydrate (Pb(CH_3_CO_2_)_2_·3H_2_O) was added in our experiments. The diffraction peak at 2*θ* = 31.35°, corresponding to the PZT (110) plane, was obviously stronger than the other peaks. The strong and sharp diffraction peaks are coincident with the peak pattern of the PZT perovskite crystalline structure [35]. Nevertheless, for the free-standing film, especially the thin film, the strain energy required to form the perovskite phase was usually diminished due to the strain relaxation in the direction perpendicular to the thin film [32, 33]. As a consequence, the existence of a surface pyrochlore phase cannot be avoided. To further confirm its composition, Raman spectrum of the nanodot arrays was analyzed. The six peaks can be recognized at 204, 273, 322, 569, 586, and 737 cm^−1^, corresponding to the lattice vibration modes of E(2TO), E_T_+B_1_, A_1_(2TO), E(3TO), A_1_(2TO), and A_1_(3LO), respectively. The observed Raman shift peaks are in accordance with the typical Raman shift peaks of the perovskite phase PZT [35-37].

### Ferroelectric Properties

**Fig. 3:**
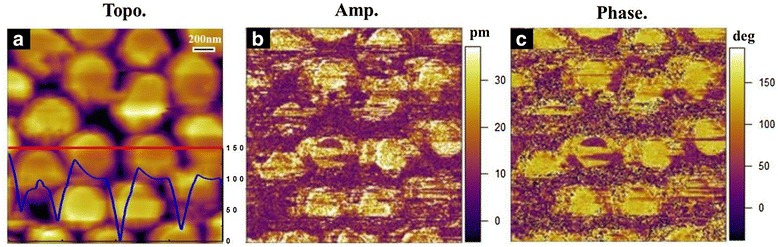
Piezoresponse images for the polarization reversal process in the nanodot arrays. **a** Topological, in which the *blue line* represents the AFM cross-sectional height data along the scanned *red line*. **b** Piezoresponse amplitude and **c** phase micrograph of the PZT nanodot arrays on a Pt/Si substrate in the same selected area

To characterize the ferroelectric properties of the ordered PZT nanodot arrays, vertical piezoresponse force microscopy (VPFM) measurements were performed and demonstrated in Fig. 3, including the AFM topography, piezoresponse amplitude, and phase micrograph. The surface topology of the PZT nanodot arrays (shown in Fig. 3a) exhibited a uniform height and well-aligned ordering. The blue line in Fig. 3a represented the AFM cross-sectional height data along the scanned red line on the PZT nanodot arrays. The average height of PZT nanodot was about 100 nm. The contrasts in amplitude piezoresponse (Fig. 3b) represent the magnitudes of the piezoelectric signals, which are much higher on the nanodots. The dark and bright areas in the phase micrograph (Fig. 3c) correspond to the up-polarization and down-polarization states, respectively, indicating the well-defined piezoresponse of the ordered PZT nanodot arrays.

**Fig. 4:**
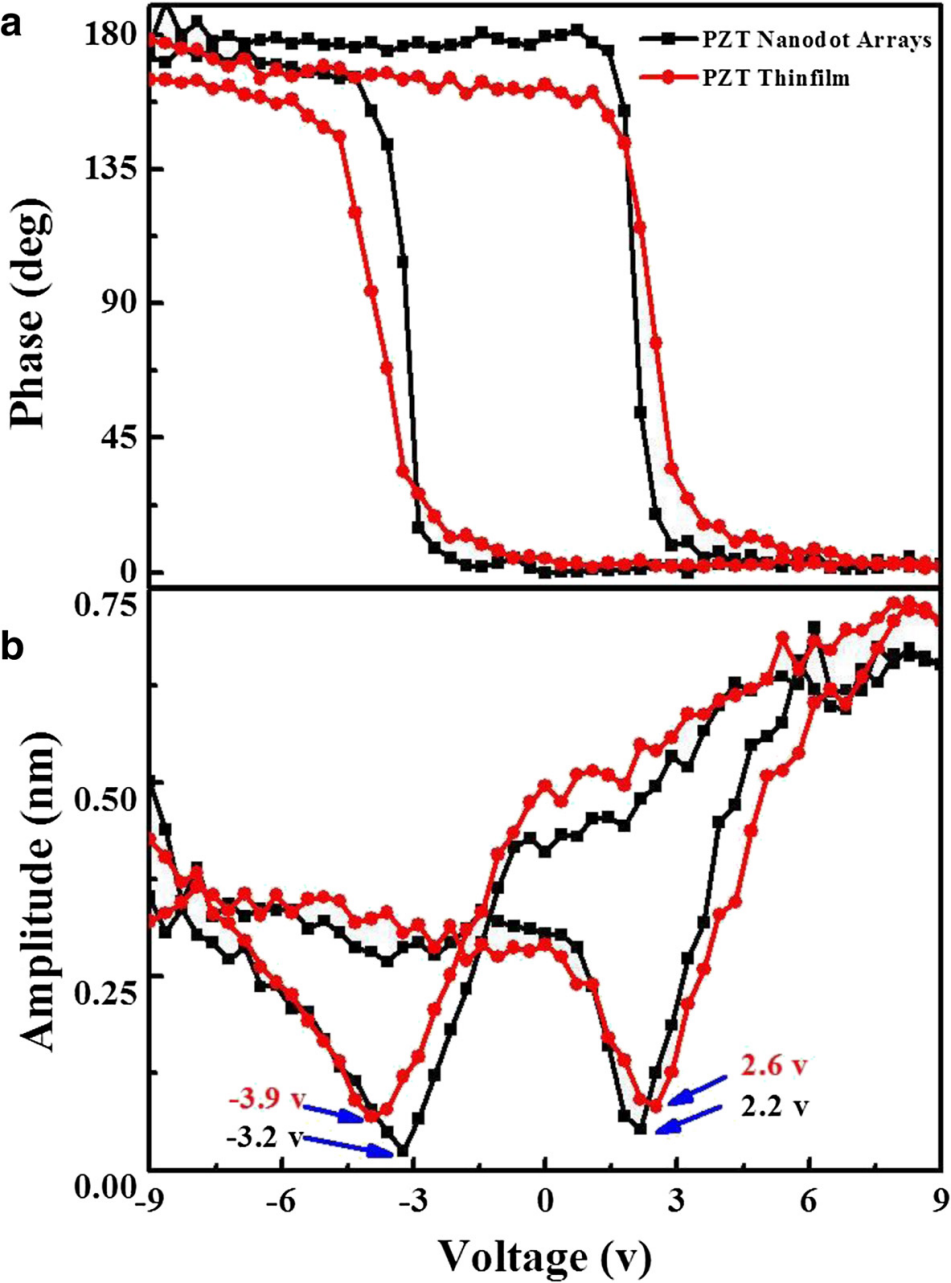
Local piezoresponse hysteresis loops acquired on a single PZT nanodot (*black lines*) and PZT thin film (*red lines*). **a** The amplitude-voltage loop and **b** the phase-voltage loop

Furthermore, to examine the local ferroelectric properties of PZT nanodots, the piezoelectric hysteresis loops on a single PZT nanodot were measured. In addition, the piezoelectric properties of the PZT thin film with the same spin coating conditions as the PZT nanodots were measured to exhibit the differences. The black lines of the piezoresponse butterfly-like amplitude-voltage loop and the phase-voltage hysteresis loop in Fig. 4a, b were on behalf of the PZT nanodot, while the red ones represented the PZT thin film. With the bias voltage increasing from +9 to −9 V, the phase change was about 175° plotted in Fig. 4a, being slightly smaller than 180°. Such a phase change indicated that the low aspect ratio of nanostructure resulted in an easier polarization switching [35]. Meanwhile, a well-developed butterfly-shaped amplitude loop and a square-shaped phase hysteresis loop can be observed in Fig. 4b. The coercive fields of the PZT nanodot were −3.2 and 2.1 V, while the ones of PZT thin film were −3.9 and 2.6 V, all indicating that the polarization reversal was asymmetric, which might be caused by the surface changes stored at the interface between PZT and Pt electrode [38]. Obviously, the coercive fields of PZT nanodot were smaller than the ones of PZT thin film, which may be caused by the release of the local field in nanodot resulting in smaller polarization reversal voltage [39]. Therefore, the PZT nanodot arrays with good polarization switching properties could act as a memory element in nonvolatile ferroelectric random access memory (NV-FRAM) devices [40].

## Conclusions

In summary, a nanopatterning strategy for the ordered PZT nanodot arrays by one-step mask etching has been presented. The ordered disconnected hemispherical nanostructures of BL played the role of etching mask rather than the conventional pore-through AAO mask. The PZT nanodot arrays on Pt/Si substrate had a well-crystallized perovskite structure and a large-area ordering. In VPFM measurements, the PZT nanodots demonstrated well-defined piezoresponse properties for a potential NV-FRAM application. The one-step mask etching strategy for ordered ferroelectric nanodot arrays is without introduction of any sacrifice layer and lithography process, being green and environmentally friendly. In addition, such a strategy can be extended to other functional materials and ordered low-dimensional multi-layered nanostructures for the kinds of nanodevice applications.

## Abbreviations

AAO: anodic aluminum oxide
AFM: atomic force microscopy
BL: barrier layer
CVD: chemical vapor deposition
FESEM: field-emission scanning electron microscope
IBE: ion beam etching
MBE: molecular beam epitaxy
NV-FRAM: nonvolatile ferroelectric random access memory
PE: plasma etching
PFM: piezoresponse force microscopy
PVD: physical vapor deposition
PZT: lead zirconate titanate Pb(Zr_0.52_Ti_0.48_)O_3_
RIE: reactive ion etching
RTP: rapid thermal process
XRD: X-ray diffraction
